# In TFIIH, XPD Helicase Is Exclusively Devoted to DNA Repair

**DOI:** 10.1371/journal.pbio.1001954

**Published:** 2014-09-30

**Authors:** Jochen Kuper, Cathy Braun, Agnes Elias, Gudrun Michels, Florian Sauer, Dominik R. Schmitt, Arnaud Poterszman, Jean-Marc Egly, Caroline Kisker

**Affiliations:** 1 Rudolf Virchow Center for Experimental Biomedicine, Institute for Structural Biology, University of Würzburg, Würzburg, Germany; 2 Institut de Génétique et de Biologie Moléculaire et Cellulaire, Centre National de la Recherche Scientifique/Institut National de la Santé et de la Recherche Médicale/Université de Strasbourg, Illkirch Cedex, France; 3 Comprehensive Cancer Center Mainfranken, University of Würzburg, Würzburg, Germany; University of California San Diego, United States of America

## Abstract

The eukaryotic XPD helicase is an essential subunit of TFIIH involved in both transcription and nucleotide excision repair (NER). Mutations in human XPD are associated with several inherited diseases such as xeroderma pigmentosum, Cockayne syndrome, and trichothiodystrophy. We performed a comparative analysis of XPD from *Homo sapiens* and *Chaetomium thermophilum* (a closely related thermostable fungal orthologue) to decipher the different molecular prerequisites necessary for either transcription or DNA repair. *In vitro* and *in vivo* assays demonstrate that mutations in the 4Fe4S cluster domain of XPD abrogate the NER function of TFIIH and do not affect its transcriptional activity. We show that the p44-dependent activation of XPD is promoted by the stimulation of its ATPase activity. Furthermore, we clearly demonstrate that XPD requires DNA binding, ATPase, and helicase activity to function in NER. In contrast, these enzymatic properties are dispensable for transcription initiation. XPD helicase is thus exclusively devoted to NER and merely acts as a structural scaffold to maintain TFIIH integrity during transcription.

## Abstract 2

The DNA helicase activity of the xeroderma pigmentosum D protein, a crucial subunit of TFIID, is only needed for its role in DNA repair, not for transcription.

## Author Summary

The multiprotein complex TFIIH is crucially involved in two fundamental cellular processes—the transcription of genes by RNA polymerase II and the repair of UV-induced DNA damage by a mechanism called nucleotide excision repair (NER). The xeroderma pigmentosum complementation group D (XPD) helicase, which is mutated in the eponymous human photosensitivity and cancer syndrome, is an important subunit of TFIIH, where it is assumed to act as a helicase, unwinding the DNA double helix. In our study, we show that XPD assumes an entirely different role in transcription and NER. In the case of repair, this protein works as an enzyme, requiring all its known functional properties. For transcription, however, none of the enzymatic functions is essential and XPD switches from an enzyme to a structural protein whose job is merely to preserve the integrity of the TFIIH complex. We have shown thus that the helicase activity of the XPD protein is exclusively devoted to repair processes and could be targeted by drugs without affecting transcription.

## Introduction

The human XPD protein is a helicase with 5′–3′ polarity [1],[2] that contains a 4Fe4S cluster (FeS) [3]–[6] and is organized within the context of the general transcription factor IIH (TFIIH). TFIIH consists of a total of 10 subunits of which XPB, p62, p52, p44, p34, and p8 build the core and cdk7, MAT1, and cyclin H comprise an additional regulatory unit called the CAK complex; both subcomplexes are bridged by XPD [7]–[10]. The first assigned function of TFIIH was its role as a basal transcription factor promoting RNA polymerase II (RNAPII)–based transcription [10]–[13]. Within this process, TFIIH participates in initiation, promoter escape, and the early elongation steps [9],[14]–[18]. Later it has been shown that TFIIH is also involved in RNA polymerase I– and probably III–based transcription [8],[19]. While the TFIIH core assumes responsibility for promoter opening, the CAK subcomplex promotes initiation and elongation through phosphorylation of the C-terminal domain (CTD) of the Rbp1 subunit of RNAPII. In addition, it has been shown that CAK is also responsible for the transactivation of nuclear receptors.

**Figure 1:**
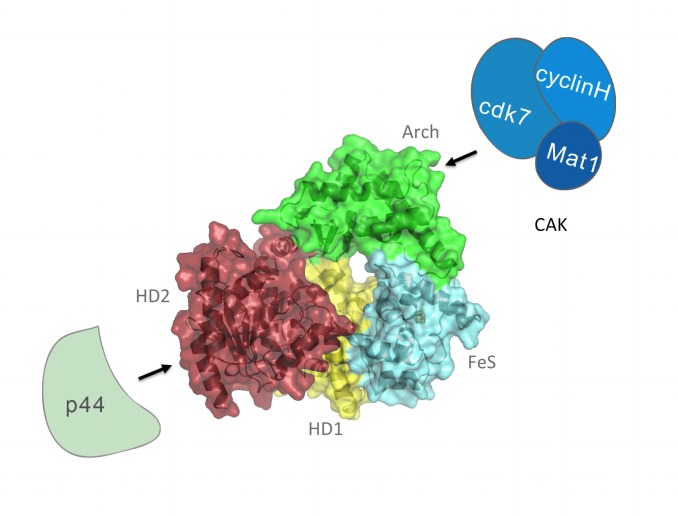
XPD and its partners in TFIIH. Schematic representation of XPD and its interaction partners p44 and MAT1 within the CAK complex of TFIIH. XPD from taXPD in the center is shown with the two helicase domains (HD1 and HD2) in yellow and red, respectively, with the FeS cluster-containing domain in cyan and the Arch domain in green. P44, which interacts with the C-terminal end of XPD, is shown in pale green The CAK complex, which interacts with XPD via the MAT1 subunit, is shown in different blue colors.

Next to its function in transcription, TFIIH plays a major role in eukaryotic nucleotide excision repair (NER) [10],[20]–[22]. NER is the most versatile DNA repair pathway due to its ability to remove bulky lesions, as well as smaller lesions such as cisplatin adducts or photoproducts caused by ultraviolet light [23]–[26]. In NER, TFIIH is recruited to the site of damage by the XPC–HR23B complex after initial damage recognition has been achieved [23],[27]–[29]. At this point, TFIIH is of vital importance for damage verification and the procession of the NER cascade resulting in the excision of the damaged DNA fragment. The significance of TFIIH within the repair cascade is reflected in its association with three severe hereditary human diseases: xeroderma pigmentosum (XP), Cockayne syndrome (CS), and trichothiodystrophy (TTD). It is interesting to note that TTD patients seem to be also affected partly in aspects of transcription [26],[30],[31]. In the context of human diseases, it is particularly noteworthy that mutations causing XP, CS, or TTD can be located on a single gene, namely XPD [1],[32], that also harbors most of the described mutations that are associated with TFIIH. It has been enigmatic for a long time how these very different phenotypes can be associated with a single gene. Recent crystal structures of archaeal XPD homologues shed some light on this puzzling matter. The structures revealed that XPD contains four domains (Figure 1), of which two are canonical RecA-like domains (helicase domains 1 and 2, HD1 and HD2), and two additional domains that are inserted in HD1, a so-called Arch domain and a domain harboring the FeS cluster [3],[5],[6],[23].

XPD as well as XPB belong to superfamily 2 (SF2) helicases but operate with opposite polarities [7],[9],[10],[33]. In addition, the roles of XPD and XPB in transcription and repair seem to differ significantly [10],[34]. The helicase activity of XPB is required for promoter opening and promoter escape after the preinitiation complex (PIC) has been assembled [9],[16],[17],[26],[35]. In NER only the ATPase activity of XPB is necessary to anchor TFIIH to the lesion site [19],[26],[32], whereas its helicase activity seems to be dispensable [10],[19],[25]. In contrast, XPD is a vital factor for NER, including its helicase activity [23],[25],[26],[36]; XPD is interacting (Figure 1) with the p44 subunit within the TFIIH core and with the MAT1 subunit of the CAK complex [23],[28],[37]. Whereas p44 binds to the very C terminus of XPD [4],[6],[26],[37],[38], MAT1 is recruited via the Arch domain [23],[32]. It has been shown that p44 is able to stimulate the helicase activity of XPD [5],[6],[23],[37] and that the interaction with CAK negatively regulates the helicase activity of XPD [33],[37]. For transcription, however, it is less clear which of the XPD activities are required. Early work in yeast has shown that a temperature-sensitive mutant of Rad3, the XPD ortholog in yeast, is affected in transcription [34],[39]. It has also been shown that TTD-inducing XPD mutations that abolish the interaction with p44 have an impact on the transcriptional properties of TFIIH [9],[22],[26]. Furthermore, mutations in the Arch and FeS domains cause a TTD phenotype and show decreased transcriptional activity [26],[32]. In contrast, the Walker A motif variant K48R, which displays no ATPase and helicase activities, supports transcription [19],[25],[26].

In this study, we have used a combinatorial approach of characterizing two eukaryotic XPD proteins to delineate which of its properties and domains are dedicated to transcription and NER. We show that the FeS domain of XPD is crucial for NER. In addition, our results clearly demonstrate that the repair process depends on XPD's biochemical and enzymatic properties for successful NER, thus requiring DNA binding, ATPase activity, helicase activity, as well as the correct interaction with other TFIIH subunits. In stark contrast, all enzymatic properties including DNA binding are not essential for the different steps in transcription; here, XPD only acts as a scaffold within TFIIH with the sole requirement to support protein–protein interactions.

## Results

### Comparative Mutagenesis of Human and Fungal XPD

Proteins from the fungus *Chaetomium thermophilum* provide an ideal basis to perform biochemical studies, as they are closely related to their human homologues but are significantly more stable and thus amenable to purification [36],[40]. We employed a comparative functional mutagenesis study of *C. thermophilum* XPD (ctXPD) and human XPD (hsXPD). The sequence alignment (Figure S1a) shows that ctXPD and hsXPD are closely related with a relative homology of 74% and an identity of 55% (as defined by Blast). Based on the sequence alignment, homology modeling, and previous studies on *Thermoplasma acidophilum* XPD (taXPD) [37],[41]–[43], we chose the following variants to be generated in hsXPD and ctXPD: hsC134S/ctC133S, hsY158A/ctY156A, hsF193A/ctF192A, hsR196A/ctR195A, hsR196E/ctR195E, and hsR722W/ctK719W, of which five residues are identical and the remaining is type-conserved. The variants include a TTD mutation (hsR722W/ctK719W) and point mutations that have been shown to be of functional significance in archaeal homologs of XPD (Figure S1a,b and Table 1). In addition, we generated the ctK48R variant, which disrupts the Walker A motif and thus ATP hydrolysis of XPD in *C. thermophilum*, to serve as a negative control. We focused on variants located in the N-terminal domain of XPD—that is, the FeS domain—as it has been implicated as being highly important for archaeal XPD function [4],[6],[28],[37],[38]. To further analyze hsXPD, we introduced three additional variants. HsL372A was generated to test whether a mutation in the Arch domain, which is most likely not involved in DNA binding, ATPase activity, and helicase activity, would affect the function of hsXPD. In addition to hsC134S/ctC133S, the variant hsC155S was designed to further probe the FeS cluster of hsXPD (both Cys134 and Cys155 coordinate the FeS), and hsF161A as an alternative residue for the corresponding ctY156A variant (see Figure S1a). None of the ctXPD variants affected its overall fold, as they could be expressed and purified to at least 95% homogeneity, with the exception of the ctC133S variant (Figure S2a) and the analysis by CD spectroscopy showed that the wild-type protein and all variants display similar CD spectra (Figure S2b).

**Table 1:** List of ctXPD and hsXPD variants, their location, and properties.

Variant in *C. thermophilum*	Variant in *H. sapiens*	Structure Element	ssDNA Binding	ATPase	Helicase	Transcription	NER
K48R	K48R	HD1	+	−	−	+	−
C133S	C134S	FeS	n.d.	n.d.	−	+	−
—	C155S	FeS	n.d.	n.d.	−	+	−
Y156A	Y158A, F161A	FeS	−	−	−	+	−
F192A	F193A	FeS	−	−	−	+	−
R195A/E	R196A/E	FeS	−	−	−	+	−
—	L372A	Arch	n.d.	n.d.	+	+	+
K719W	R722W	HD2	+	−	−	−	−

−, indicates impaired activity; +, activity; n.d., not determined.

### Interaction of ctXPD with ctp44

**Figure 2:**
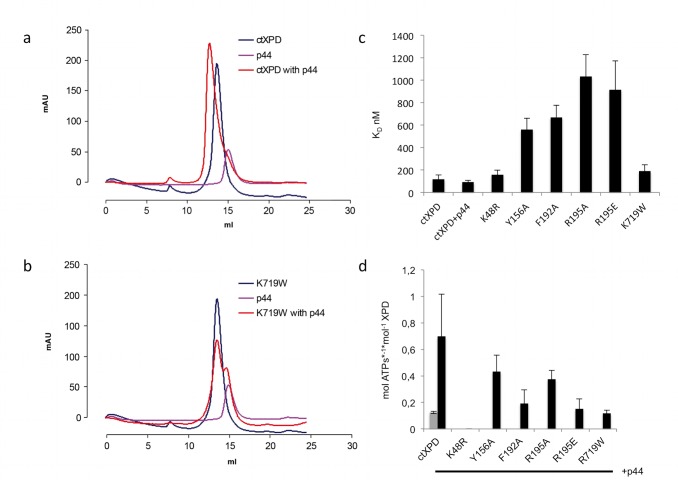
Functional analysis of ctXPD. (a) SEC of ctXPD wild type with ctp44 (1–285). Ctp44 (pink) and ctXPD (blue) were analyzed separately and in a 1∶1 stoichiometry (red) mixed prior to SEC. (b) SEC of the ctK719W variant with ctp44 (1–285). The color coding is chosen as in (a). (c) ssDNA binding of ctXPD was analyzed by biolayer interferometry. Measurements were performed in triplicate and with different protein batches. (d) *In vitro* ATPase assay of ctXPD wild type in the presence (black bars) and absence of ctp44 (1–285) (grey bar). Subsequently all variants were analyzed in the presence of ctp44 (1–285). The data used to generate panels (c) and (d) have been deposited as supplementary information in xls format (Table S1).

To fully exploit our ctXPD model system, we also cloned and expressed *C. thermophilum* p44 (ctp44) comprising residues 1–285. This construct is analogous to an hsp44 construct that is fully capable of activating hsXPD [8],[23] but lacks the C-terminal Ring and Zn-finger domains of p44. Size exclusion chromatography (SEC) experiments revealed that equimolar ratios of ctXPD and ctp44 form a stable complex exemplified by a significant shift in the elution volume of a single peak representing the ctXPD–ctp44 complex, which can be clearly distinguished from the peaks of the single proteins (Figure 2a). In contrast, the ctR719W variant, which corresponds to the human R722W variant that abrogates the p44 interaction with hsXPD, displays no shift, thus clearly indicating the impairment of complex formation (Figure 2b). We then investigated all ctXPD variants for complex formation with ctp44 and observed ctXPD/ctp44 wild-type–like complex formation for all other variants, thus indicating that none of the other mutations interfered with p44 interaction (Figure S2c).

### ssDNA Binding of ctXPD Variants

We assessed the ssDNA binding properties of ctXPD and its variants (Figure 2c and Table 2). The wild-type protein displayed a dissociation constant (K_D_) of 118 nM, which was not altered in the presence of p44 (95 nM); hence, we omitted p44 from the analysis of the variants. The affinity of ctXPD to ssDNA is very comparable to the affinity of the archaeal homologues [5],[6],[37],[38],[44]. The ctK48R and ctK719W variants display no major alteration in affinity for ssDNA with K_D_ values of 159 nM and 192 nM, respectively. Because the ctK48R variant contains a mutation in the helicase Walker A motif that is solely responsible for ATP hydrolysis, the results are in line with the role of this motif. The ssDNA affinity of ctK719W clearly shows that DNA binding is not affected, implying that the deficiency to interact with p44 impairs XPDs helicase activity by other means; this is in line with the fact that p44 does not influence the affinity of ctXPD for ssDNA. All other ctXPD variants are significantly impaired with respect to their ability to bind to ssDNA, with K_D_ values ranging from 561 nM to 1,035 nM (Table 2), which is in agreement with their proposed proximity to the DNA path along the XPD molecule [37],[38].

**Table 2:** Biochemical parameters of ctXPD and variants.

Variant	K_D_ (nM) ssDNA	ATPase Activity XPD (mol ATP_·_s^−1^_·_mol^−1^ XPD)	K_D_ (nM)+p44 ssDNA	ATPase Activity XPD+p44 (mol ATP_·_s^−1^_·_mol^−1^ XPD)	Helicase Activity+p44 (Rel. Fluorescence Change·s^−1^)
Wild type	118±37	0.12±0.01	93±16	0.70±0.32	1,906.3±408.9
K48R	159±40	-	-	n.d.	7.8±6.7
Y156A	561±100	-	-	0.43±0.12	407.5±70.1
F192A	668±107	-	-	0.19±0.10	24.1±7.1
R195E	916±257	-	-	0.15±0.07	9.3±7.2
R195A	1,035±193	-	-	0.38±0.07	11.2±4.3
K719W	192±54	-	-	0.12±0.02	4.8±2.3

### ssDNA-Dependent ATPase Activity of ctXPD Variants

Due to the possibility to express functional fungal XPD and p44 separately, we first analyzed whether the ctXPD ATPase activity is dependent on p44 activation. Surprisingly, we observed a clear p44-related effect. The activity increased from 0.12 mol ATP·mol XPD^−1^·s^−1^ to 0.7 mol ATP·mol XPD^−1^·s^−1^ after adding ctp44 to ctXPD in a 2∶1 molecular ratio (Figure 2d and Table 2). Because the ssDNA affinity of XPD itself is not altered by p44 binding, p44 must directly activate the ATPase function of XPD. As expected, the ctK48R Walker A variant is ATPase deficient. The decreased affinity for ssDNA in the ctY156A variant leads to a reduced activation of the ATPase activity, with 61% of wild-type activity. Surprisingly, the ctF192A variant that bound to ssDNA with a 6-fold increase in K_D_ (Figure 2c) only displayed 27% of wild-type ATPase activity. Inferred from the structures of archaeal XPDs [37], this residue is not involved in ATP binding; thus, F192 may participate in an ATP hydrolysis-mediated helicase step on the translocated ssDNA using base stacking interactions [4]–[6],[39]. The impaired translocation on ssDNA could disturb the concerted action of HD1 and HD2 during ssDNA-dependent ATP hydrolysis and provides a likely explanation for its strongly reduced ATPase activity. The two variants R195E/A displayed an approximately 10-fold decrease in affinity for ssDNA. Whereas the ATPase activity of R195A was only moderately affected with a decrease to 54% compared to wild-type ctXPD, ctR195E is even further reduced and displayed only 20% residual activity. The p44 interaction-deficient ctK719W variant displayed a highly decreased ATPase activity reflecting the basal ctXPD ATPase level in the absence of p44, thus further supporting the notion that p44 directly stimulates XPD's ATPase activity. All other variants displayed a significant decrease in p44-dependent ATPase activity, which can be explained by their reduced DNA binding ability, as ssDNA is necessary to trigger XPD's ATPase activity.

### Helicase Activity of XPD Variants

**Figure 3:**
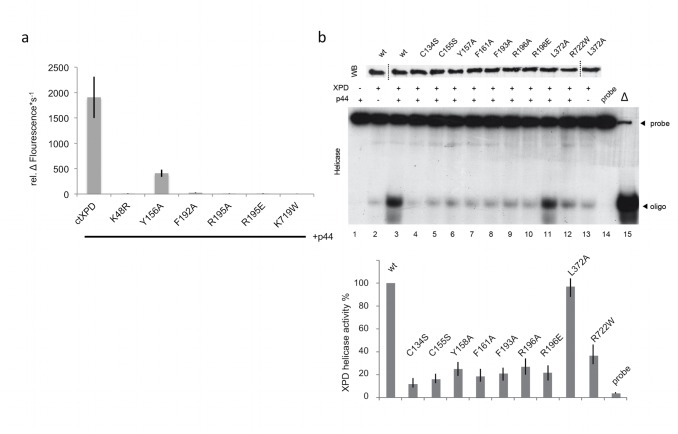
Helicase activity of ctXPD and hsXPD and its variants. (a) Helicase activity of wild-type ctXPD in the presence of ctp44 (1–285) and its variants analyzed utilizing a fluorescence-based helicase assay. (b) hsXPD wild-type and variants were expressed in insect cells using the baculovirus overexpression system and immunoprecipitated using an antibody (Ab) directed toward the Flag epitope fused at the N terminus of the protein. After elution with the Flag synthetic peptide, equal amounts of purified proteins were resolved by SDS/PAGE with 12% (w/v) polyacrylamide followed by Western blot (WB) analysis (upper panel, lanes 3 to 12). Bands shown in lanes 2 and 13 in the upper panel are a duplication of lanes 3 (wild type) and 11 (L372A). Purified XPD wild type and variants were added to a 5′-strand extension probe in the presence of an excess of p44 to evaluate their 5′–3′ helicase activity. The reaction was analyzed by electrophoresis on a 14% (w/v) polyacrylamide gel and analyzed by autoradiography. Controls include reactions performed in the absence of wild-type XPD (lane 1) and in the absence of p44 (lane 2). The native and denatured probes have also been analyzed (lanes 14 and 15, lower panel). The data used to generate panels (a) and (b) have been deposited as supplementary information in xls format (Table S2).

To investigate the influence of the XPD mutations on the helicase activity of XPD, we performed *in vitro* helicase assays with ctXPD and hsXPD in the presence and in the absence of p44 (see Materials and Methods, Figure 3a,b, and Table 2). In the absence of ctp44, no significant unwinding by ctXPD could be detected. In the presence of ctp44, wild-type ctXPD was readily unwinding the 5′ overhang substrate and yielded an activity of 1,906.3 ΔFl.·s^−1^, indicating a robust 5′–3′ polarity (Figure 3). The walker A motif mutant ctK48R, which is unable to hydrolyze ATP, also failed to separate dsDNA (7.8 ΔFl.·s^−1^). The ctK719W variant was also highly affected in its helicase activity (4.8 ΔFl.·s^−1^), due to its loss of p44 interaction, resulting in a highly decreased ATPase activity. The ctF192A and ctR195A/E variants that were impaired in DNA binding and ATPase activity were highly deficient with respect to their p44-dependent helicase activity, with values of 8.6 ΔFl.·s^−1^, 24.1 ΔFl.·s^−1^, 9.3 ΔFl.·s^−1^, and 11.2 ΔFl.·s^−1^, respectively. Because the loss of helicase activity cannot be explained by the lack of p44 interactions, these data substantiate the role of these residues in DNA binding, which subsequently affects the ATPase and helicase activities of XPD. The only variant displaying notable p44-dependent helicase activity was ctY156A, with an approximately 5-fold reduction in activity, thus still being significantly impaired.

We analyzed the helicase activity of the hsXPD variants with an *in vitro* assay that detects the displacement of a ^32^P-labeled DNA fragment previously annealed to a single-stranded circular DNA [22],[26]. Upon interaction with p44, hsXPD exhibited a high and significant 5′–3′ helicase activity (Figure 3b, lanes 2–3 and the histogram below), whereas the hsR722W variant was not stimulated by p44 (lane 12) due to the loss of p44 interaction (Figure S3c) [23],[26], as also observed for the corresponding proteins from *C. thermophilum* (Figure 2b). All other variants displayed a strongly diminished helicase activity. Notably, hsF161A, which was chosen as a possible alternative for ctY156A, showed highly impaired helicase activity, indicating its functional importance. Curiously, hsC134S, the counterpart to ctC133S, seems to behave more stably than its fungal homologue, which exhibited an altered expression profile (Figure S2a). However, even though the expression behavior of hsC134S was comparable to wild-type hsXPD, it displayed no helicase activity, which is most likely caused by affecting the overall stability of the FeS domain of hsXPD. A similar explanation can be assumed for the other variant targeting the FeS cluster, hsC155S, which also abolished helicase activity. The only exception to the loss-of-function variants was hsL372A, which displayed wild-type–like helicase activity, confirming that this residue is not directly involved in helicase activity.

Importantly, the helicase phenotypes observed for the hsXPD variants are highly comparable to the phenotypes of the ctXPD variants. Due to the strong correlation of the helicase phenotypes in the human and the fungal system as well as the interaction studies with p44 and the CAK subunits (see below), it is valid to assume that the defects causing the deficiency in helicase activity by the lack of ssDNA binding, ATPase activity, or p44 interaction can be directly translated from ctXPD to hsXPD.

### NER Properties of hsXPD Variants

**Figure 4:**
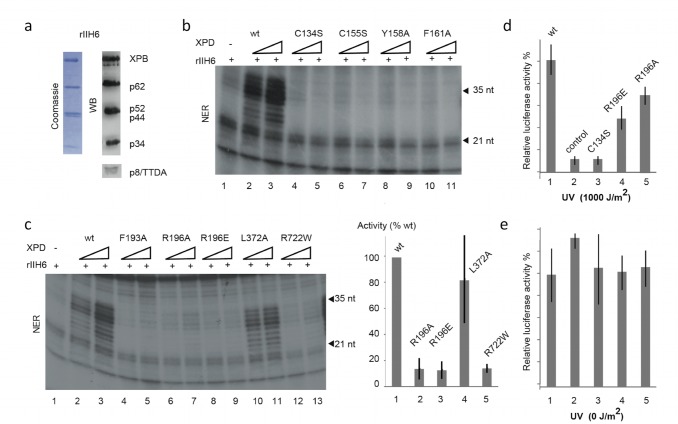
NER activity of hsXPD and its variants. (a) Purified core-TFIIH (rIIH6) resolved by SDS-PAGE followed by Coomassie staining and Western blot analysis. (b and c) NER activity of reconstituted TFIIH containing mutant XPD proteins. XPD wild type or variants (100 and 200 ng) were mixed with purified core-TFIIH (rIIH6) and added to an *in vitro* double-incision assay using recombinant NER factors. The reaction was analyzed by electrophoresis followed by autoradiography. Incision activities from three independent experiments were quantified and normalized to wild type. (d and e) Host cell reactivation activity of XPD variants. HD2 fibroblasts were transfected with a reporter plasmid expressing firefly luciferase previously exposed to 1,000 J/cm^2^ UVC-light (254 nm) (d) or with the nonirradiated control (e) in combination with vector expressing renilla luciferase to normalize transfection efficiencies and pIERS2-EGFP expressing XPD wild-type or mutant proteins. The firefly luciferase activity in cell lysates (48 h posttransfection), normalized with the internal renilla luciferase standard, assesses repair complementation. The values of three independent experiments are presented as percentages, with 100% being the level of luciferase activity obtained with wild-type XPD. The data used to generate panels (c), (d), and (e) have been deposited as supplementary information in xls format (Table S3).

To assess the ability to perform NER *in vitro*, a dual incision assay was used in which hsXPD or its variants were added to purified recombinant human core-TFIIH (rIIH6) (Figure 4a), together with XPC-HR23B, XPA, RPA, XPG, ERCC1-XPF, and a closed circular plasmid with a single 1,3-intrastrand d(GpTpG) cisplatin-DNA crosslink [25],[26],[34]. In the absence of hsXPD, the XPG and ERCC1-XPF endonucleases were unable to excise the damaged DNA and liberate the damaged oligonucleotide with a length of 23–25 nt (Figure 4b,c, compare lanes 1 to lanes 2 and 3). All investigated hsXPD variants, with the exception of the L372A variant, lacked the ability to catalyze successful NER within the reconstituted rIIH complexes (Figure 4b,c). The activity of the variants is comparable to that of the hsR722W mutation (Figure 4c, lanes 12 and 13 and histogram). HsL372A behaves indistinguishably from wild-type XPD, further supporting that this residue is not relevant for NER activity.

A host cell reactivation assay was used to investigate the *in vivo* repair capabilities of selected XPD variants. In this assay, the capacity of XPD variants to repair and express a damaged gene in a cellular context utilizing HD2 cells is measured. HD2 cells are NER-deficient, resulting from the fusion between human fibroblasts harboring the XPD/R683W point mutation and HeLa cells [32],[40]. A plasmid encoding the firefly luciferase gene was exposed to UVC radiation and then transfected in combination with a second vector expressing Renilla luciferase to normalize transfection efficiencies. A third vector expressing the XPD variants was added to the cells (Figure 4d,e). The firefly luciferase activity in cell lysates was measured 48 h posttransfection and normalized using wild-type activity as a reference. Transfection of hsC134S, which exhibited almost no *in vitro* double incision activity, did not permit the expression of the luciferase gene after UV irradiation and exhibited an activity comparable to that of the control transfected with an empty vector (Figure 4d, lanes 2 and 3). However, hsR196A/E, which also showed reduced *in vitro* double incision activity, partially permitted the expression of luciferase. The nonirradiated control experiments showed a comparably high expression activity in all cases (Figure 4e). These data demonstrate that the XPD variants are defective in DNA repair both *in vitro* and *in vivo*. The expression of the luciferase in the control experiment without UV irradiation was not affected, indicating regular transcription and expression promoted by the XPD variants. To exclude the possibility that the negative effects in repair are due to a disturbed CAK interaction, we performed interaction studies of wild-type ctXPD and its variants with ctMAT1 (residues 1–248) employing native PAGE experiments. Figure S3a shows that all variants form a complex with ctMAT1 in a wild-type–like manner, indicating no impairment in CAK interaction (Figure S3a). In a parallel approach, we probed the interaction of p44 and CAK with hsXPD, hsC134S, hsR722W, and hsK48R using pull-down experiments (Figure S3b,c). Our data show that hsC134S and hsK48R still interact with p44 and the CAK complex. This is not the case when looking at hsR722W, which shows an impairment in p44 interaction but not in CAK interaction. Our data thus exclude the possibility that an interruption between XPD and the CAK subcomplex leads to the altered NER phenotype of the investigated XPD variants and the pull-down assays with p44 further signify the similarity between hsXPD and ctXPD

### *In Vitro* Transcriptional Activity of hsXPD Variants

**Figure 5:**
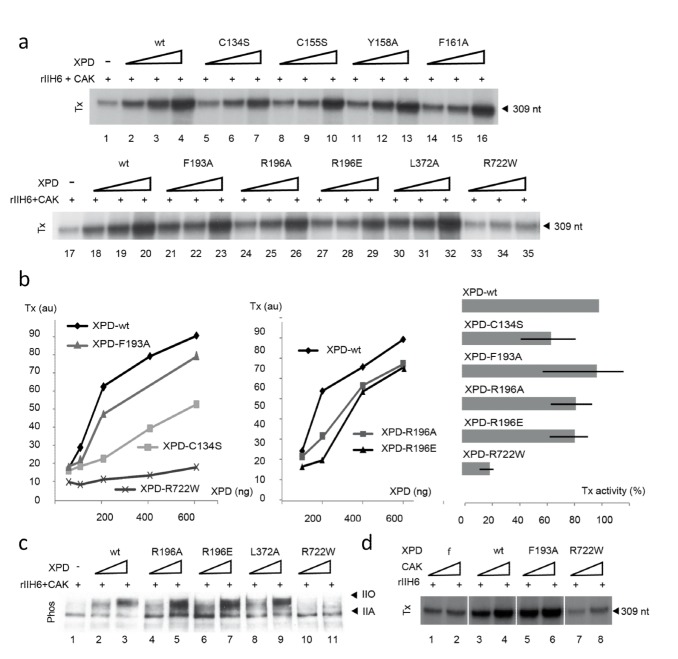
Transcriptional activity of hsXPD and its variants. (a) Basal transcription activity. Increasing amounts of the recombinant XPD variants (∼50, 100, and 250 ng) were mixed with purified core-TFIIH (rIIH6) (250 ng) and CAK (300 ng) to an *in vitro* reconstituted transcription system containing all the basal transcription factors and the AdMLP. Transcripts were analyzed by electrophoresis followed by autoradiography. The length of the corresponding transcript is indicated on the right side. (b) Dose–response curves for XPD-wt and mutants C134S, F193A, R196A/E, and R722W. We have selected the hsF193A and hsR196A/E variants that exhibited levels of transcription similar to wild-type hsXPD as a reference. The transcription activity was estimated from densitometric analysis of autoradiograms (arbitrary units). The data are also plotted as % wild-type activity in the panel to the right. (c) CTD phosphorylation of RNAP II by reconstituted TFIIH with XPD variants was analyzed by SDS-PAGE and followed by WB detection. Purified core-TFIIH (250 ng), CAK (300 ng), and XPD variants (100 or 300 ng) were mixed in an *in vitro* assay containing all the basal transcription factors and the AdMLP. Arrows indicate hypophosphorylated (IIA) and hyperphosphorylated (IIO) forms of RNAP II. (d) Transcription activity of reconstituted TFIIH containing XPD variants (250 ng core-TFIIH and 200 ng XPD) was assessed in the presence of different amounts of purified recombinant CAK (150 and 300 ng).

We next investigated the transcriptional properties of the rIIH6 complexes in an *in vitro* reconstitution assay in which XPD variants were added in increasing amounts to purified rIIH6 core and CAK in addition to the recombinant basal transcription factors TBP, TFIIA, TFIIB, TFIIE, TFIIF, as well as purified endogenous RNAP II (Figure 5a). Surprisingly, most hsXPD variants led to a transcriptional activity comparable to wild-type XPD in terms of transcript length and amount, regardless of their enzymatic impairment. The exceptions are the hsR722W (activity of 20%±3%) and, to a much lesser extent, the hsC134S (activity of 65%±18%) variants. The transcriptional defect of hsR722W (Figure 5a, lanes 33–35 and Figure 5b) can readily be explained due to the lack of an interaction with p44 (Figure S3c). This mutant also displayed the highest impairment of all variants. HsC134S most likely constitutes a protein with a compromised FeS domain that could affect its overall stability. Titration experiments show that the effect on transcriptional activity of hsC134S is only moderate as compared to other variants or hsXPD (Figure 5b). However, although the FeS domain is compromised, the protein clearly supported transcription, indicating that the FeS domain is not vital for the transcription function of XPD. In order to exclude that residual helicase activity of some XPD variants is responsible for transcriptional activity, we performed time course transcription experiments with selected hsXPD variants. The data show that wild type and K48R show a similar increase over time, hsC134S shows a slightly affected activity, whereas the hsR722W variant displays strongly reduced activity (Figure S4). Because the Walker A mutant K48R and the hsC134S variant only display background levels of helicase activity but remain active in transcription, an effect of residual helicase activity on transcription can be excluded.

To substantiate our results, we further analyzed the hyperphosphorylation state of the carboxy terminal domain (CTD) of the largest subunit (Rbp1) of RNAP II by the CAK subcomplex of TFIIH that is supposed to be anchored to the core through an interaction with XPD (Figure 5c), an important requirement for the transition between initiation and promoter escape [3],[5],[6],[41]–[43]. The hyperphosphorylated form of RNAP II (IIO) was prevalent in all variants and comparable to wild-type hsXPD, demonstrating that RNAP II was capable of elongating normally. The only exception here is the hsR722W variant that displayed nearly no hyperphosphorylation activity (Figure 5c, lanes 10 and 11). Lastly, we analyzed the effect of increasing concentrations of the CAK complex on the *in vitro* transcription activity (Figure 5d). To this end, we selected the variants hsF193A and hsR722W. While increasing amounts of the CAK complex stimulated the activity of wild-type hsXPD and hsF193A, this stimulation was substantially reduced when no XPD was present (Figure 5d, lanes 1 and 2). A similar phenotype was observed for hsR722W, which also showed a significantly reduced transcription activity and increasing amounts of CAK led to similar results, as observed in the absence of XPD (Figure 5d, lanes 7 and 8). Because the hsR722W variant is not impaired in the CAK interaction (Figure S3b) [28],[32], the recruitment of CAK by XPD must be disturbed due to the lack of p44 interaction (Figure S3c), which impairs the incorporation of XPD into the core TFIIH. This effect cannot be rescued by addition of CAK in excess.

## Discussion

Several studies have underlined the dual function of TFIIH in both transcription and DNA repair (reviewed in [8]). However, these very distinct tasks may require different roles/functionalities for each of the 10 subunits and, in particular, for the two ATP-dependent helicases XPB and XPD. We here focus on XPD and its involvement in both processes, and demonstrate its specific and exclusive role as an enzyme in DNA repair.

We chose a combinatorial approach that allowed us to dissect functions of XPD by assessing the influence of the introduced variants on the primary enzymatic properties such as DNA binding, ATPase activity, helicase activity, and, in addition, on protein–protein interactions that are important for enzymatic function. In a second step, these variants were investigated in NER and transcription, to establish a relationship between a phenotype and its underlying molecular cause in a TFIIH-dependent context. We introduced mutations that especially cover the FeS domain of XPD (Figure 1 and Figure S1), as this domain has been proposed to be of importance for XPD function [37],[38],[44]. In addition, we have chosen the disease-related variant R722W located at the C terminus of XPD that disrupts the interaction with p44 and lastly the Walker A motif variant K48R. Our analysis shows that ctXPD and hsXPD are highly comparable with respect to their particular enzymatic properties and that results obtained with ctXPD can be readily extended to hsXPD. Therefore and for reasons of simplicity, we will only mention the human XPD numbers of all analyzed variants from here on.

**Figure 6:**
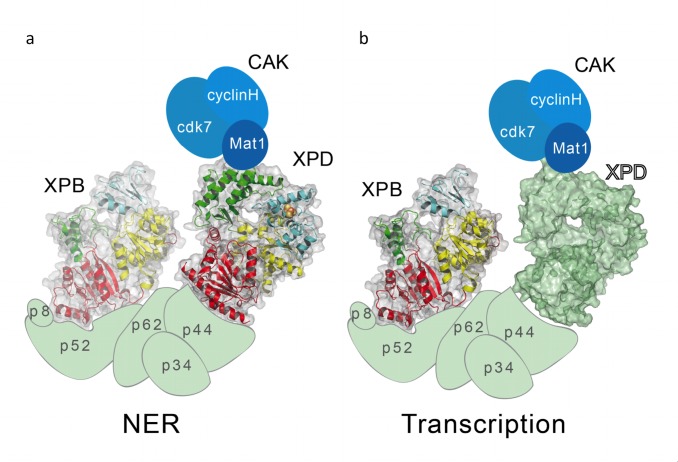
Model of the different XPD and XPB functionalities in NER and transcription. (a) In NER, XPD and XPB act as enzymes and have to mediate protein–protein interactions. XPD [3],[27],[29] and XPB [30],[31],[50] are shown as a cartoon with their molecular surface. The other subunits are depicted schematically. (b) As in (a), with the exception that the cartoon for XPD has been omitted and XPD is shown in green to exemplify that only the “shell” of XPD is needed for transcription.

All variants located in the FeS domain of XPD are impaired in helicase activity, which is caused by different defects in singular properties such as reduced DNA binding, ATPase activity, or the inability to separate dsDNA. The highly altered DNA binding ability observed in the R196E variant therefore leads subsequently to diminished ATPase and helicase activity. An interesting phenotype is, however, constituted by the Y158A variant. Although it retains 61% ATPase activity compared to wild-type XPD, the helicase activity is affected more than one would anticipate based on the ATPase data. The position of this variant in the structural model (Figure S1b) suggests that it could be involved in duplex separation, which is predicted to take place at the outer rim of the FeS domain [37],[38]. The observed lack of duplex unwinding would be a direct result of the inability to separate dsDNA, thereby targeting the helicase function of XPD without a major effect on ATPase activity. Because F161A is also highly diminished in helicase activity and is located in close proximity to Y158, it is tempting to speculate that a similar phenotype for these variants is the likely explanation. Consequently, the C134S and C155S variants directly targeting the coordination of the FeS cluster in the FeS domain are also impaired in their helicase activity, which is most likely due to unfolding of the entire FeS domain [4]–[6]. Since the structural part responsible for the separation of dsDNA is located in the FeS domain, the unfolded FeS domain cannot mediate the separation of dsDNA, resulting in an inactive helicase. In contrast, the Walker A K48R variant interacts with DNA like the wild-type protein but lacks ATPase activity and thereby causes an impaired helicase activity. The p44 interaction-deficient R722W is helicase-deficient due to an impaired interaction with p44. So far, however, it has not been known how p44 activates XPD. We show here that p44 strongly activates the ATPase activity (and consequently the unwinding activity) of XPD. This effect is not mediated by an increase in affinity for ssDNA but through a direct up-regulation of the ATPase motor activity of XPD via a yet unknown mechanism. However, the lack of p44 interaction impacts not only XPD's helicase activity but also the recruitment of XPD to TFIIH, thereby intensifying its phenotype [26]. All variants that fail to separate dsDNA for the reasons explained above are also impaired in NER. Our data thus show that it is essential for XPD to maintain its capability to act as a classical helicase in NER and that it is involved in unwinding the duplex around the lesion promoting downstream processes that lead to a successful excision (Figure 6). It is thus clear that the two helicase domains (HD1 and HD2) are essential, but our data demonstrate that the investigated FeS domain of XPD is also crucial for its enzymatic function and subsequently for NER.

We utilized the available “molecular toolkit” of variants to further dissect the involvement of XPD in transcription initiation in a TFIIH and CAK context. To our great surprise, most of the variants (C134S, C155S, Y158A, F161A, F193A, and R196A/E) supported transcription in a wild-type–like manner (Figure 5). However, it remains possible that transcription could be affected in cells, perhaps at a subset of genes, because of auxiliary factors whose activity or association with TFIIH is compromised by an XPD mutation.

Our data demonstrate that none of the enzymatic properties of XPD—namely, DNA binding, ATPase activity, and helicase activity—are relevant for this process. Even the R196E/A variant, which is highly impaired in DNA binding, has no impact on *in vitro* transcription or CTD phosphorylation (Figures 2 and 5), indicating that XPD does not interact with the DNA during transcription. This observation is further supported by recent EM structures from the PIC [45],[46] in which XPD was modeled in positions where it is not in close proximity to DNA. However, this leads to the question of which function XPD is required to fulfill during transcription, as its presence is undoubtedly important [25],[26],[34]. This can be addressed with the phenotype of the hsR722W variant, which does not support transcription. We show that this variant is neither impaired in its tertiary structure nor in DNA binding; hence, the lack of ATPase and helicase activity of this variant must be explained by the loss of p44 interaction. Taken together with the fact that the enzymatic activities of XPD are obsolete in transcription, these observations suggest that the decisive phenotype for transcription in this variant is exclusively the lack of an interaction with p44.

This lack of interaction most likely results in a misplaced XPD regarding the TFIIH composition and subsequently also the displacement of the CAK complex, as XPD not only interacts with the core-TFIIH subunit p44 but also provides the bridge to the MAT1 subunit of the CAK complex via its Arch domain (Figure 1) [32]. It has been shown that the interaction with CAK is not interrupted in the hsR722W variant (Figure S3b) [26],[32]; thus, the lack of CTD phosphorylation and transcriptional activity in our studies must be attributed to the wrong positioning of the CAK complex caused by the impaired XPD/p44 interaction. This is underlined by the observation that the hsR722W variant displays a phenotype comparable to a TFIIH lacking XPD in its entirety and cannot be rescued by increasing amounts of CAK (Figure 5d). The data therefore suggest that the essential role of XPD during transcription is to anchor the CAK to the core TFIIH and consequently support its function within the PIC. The phenotype of other TTD mutants strongly supports this hypothesis. From the X-ray structures of XPD, it was speculated that mutations, which alter the overall stability of XPD, exhibit a TTD phenotype. This includes R112H, C259Y, R592P, and D673G of human XPD [3],[5],[6]. C259Y is located in the Arch domain of XPD, and it was recently shown that it influences the CAK interaction [32], thus being the probable cause for the transcriptional defect of this variant. We therefore propose that XPD only acts as a scaffold for the structural integrity within the core TFIIH and is required for the recruitment of other enzymatic factors like the CAK. When the XPD/p44 interactions, on the one hand, and the XPD/MAT1 interactions, on the other hand, are permitted, XPD promotes transcription, despite being completely inactive as an enzyme (Figure 6). This is further supported by the phenotypes of the C134S and C155S variants that presumably comprise an unfolded FeS domain. Because the two major protein–protein interaction sites in XPD reside in the C-terminal HD2 and in the Arch domain (Figure 1), the FeS domain does not seem to be vital for either of these interactions as these two variants still support transcription. The observed reduced transcriptional activity is most likely due to an overall effect on protein stability originating from the loss of a folded FeS domain, as also proposed for the R112 TTD mutant [2],[4]. Thus, our data indicate that the FeS domain of XPD seems to be uniquely dedicated to the helicase function in NER and is dispensable for transcription. Intriguingly, the special role of the FeS cluster for NER is further supported by other studies that link this domain specifically to damage recognition [4],[44],[47],[48].

In summary, our data demonstrate that the tasks of XPD within transcription and repair are strikingly different. In NER, XPD has to fulfill several roles by promoting protein–protein interactions and acting as a helicase, whereas for transcription only the protein–protein interactions are vital. In contrast to XPB, which acts as an enzyme during NER and transcription, XPD does not assume a dual role as an enzyme (Figure 6). The enzyme XPD is vital for NER, whereas for transcription only the intact “shell” of XPD is sufficient.

Moreover, the present data allow the design of a TFIIH variant that is solely active in transcription, with repair being abrogated. In this aspect, it will be tempting to design an animal model with such a mutation that would certainly represent a pure form of XP and would be greatly useful for a better analysis of the disease itself. Moreover, in cancer research, this represents an ideal model for drug design.

## Materials and Methods

### Mutagenesis, Expression, and Purification of ctXPD, ctp44, and ctMAT1

The gene encoding full-length ctXPD, ctp44 (1–285), and ctMAT1 (1–248) were cloned from a cDNA library from *C. thermophilum* (provided by Ed Hurt). ctXPD and ctp44 were cloned in pETM11 (EMBL-Heidelberg). CtXPD mutants were generated using the Quick-Change site-directed mutagenesis kit (Stratagene). The reactions were carried out as suggested by the manufacturer's instructions. All mutants were verified by double-stranded sequencing. CtXPD wild type, the variants, and p44 were expressed as N-terminally His-tagged proteins in *Escherichia coli* BL21-CodonPlus (DE3)-RIL cells (Stratagene) by induction with 0.1 mM isopropyl-β-thiogalactoside at 14°C for 18 h. The proteins were purified to homogeneity by metal affinity chromatography (Ni-NTA, Invitrogen) followed by anion exchange chromatography (AEC) in the case of ctXPD or by SEC where indicated. AEC was performed using a MonoQ 5/50 GL column (GE Healthcare) with 50 mM NaPO_4_ pH 7.5, 1 M NaCl, and 1 mM TCEP as elution buffer. The final buffer after AEC was NaPO_4_ pH 7.5, 200 mM NaCl, and 1 mM TCEP. SEC was performed using a HiLoad 26/60 Superdex 200 prep grade column (GE Healthcare) in 20 mM Tris-HCl or HEPES (pH 8.0), 200 mM NaCl, and 1 mM TCEP. The proteins were concentrated to 5 mg/ml based on their calculated extinction coefficient using ProtParam (SwissProt) and then flash frozen for storage. In the case of ctMAT1, recombinant expression of the N-terminally thioredoxin-His-tagged fusion protein was carried out in *E. coli* BL21(DE3) star (Invitrogen) pRARE2 cells (Novagen) in TB-medium, supplemented with 50 µg/ml Zinc acetate, by addition of 0.3 mM IPTG for 16 h at 18°C. The protein was purified by IMAC (Ni-IDA, Macherey-Nagel) and dialyzed overnight in the presence of HRV-14 3C protease against 20 mM Hepes pH 8.0, 0.8 M NaCl, and 7 mM ß-Mercaptoethanol. SEC was performed as outlined above.

### ssDNA ctXPD Biolayer Interferometry Binding Assay

Real-time binding assays between ssDNA (5′-gactacgtactgttacggctccatctctaccgcaatcaggccagatctgc-3′) and purified ctXPD wild type, variants, and ctp44 were performed using biolayer interferometry on an Octet RED system (Fortebio, Menlo Park, CA). This system monitors interference of light reflected from the surface of a fiber optic sensor to measure the thickness of molecules bound to the sensor surface. The 3′-biotinolyted DNA was obtained from Biomers and coupled to kinetics grade streptavidin biosensors (Fortebio, Menlo Park, CA) at a concentration of 100 nM. Sensors coated with ssDNA were allowed to bind ctXPD in reaction buffer (20 mM Tris-HCl pH 8.0, 150 mM NaCl, 10 mM MgCl_2_, 1 mM DTT, and 1 mg/ml BSA) at different ctXPD concentrations ranging from 0.5 to 5 µM. If ctp44 was added, it was always added using a molar ratio of 1∶1 with respect to ctXPD. Measurements were carried out in triplicates and with different protein batches. Binding kinetics were calculated using the Octet Data Analysis Software 6.3, with a 1∶1 binding model, to calculate the association rate constants. Binding affinities were calculated as the ratio of dissociation and association rate constants.

### *In Vitro* ATPase Assay

CtXPD ATPase activity was measured with an *in vitro* ATPase assay in which ATP consumption is coupled to the oxidation of NADH via pyruvate kinase and lactate dehydrogenase activities. Activities were measured at 37°C in 100 µl solution, containing 1.5 U pyruvate kinase, 1.9 U lactate dehydrogenase, 2 mM phosphoenolpyruvate, and 0.15 mM NADH, 10 mM KCl, 1 mM MgCl_2_, 1 mM TCEP, and 20 mM Tris-HCl (pH 8.0). ssDNA (5′-gctcgagtctagactgcagttgagagcttgctaggacggatccctcgagg-3′) was added at a final concentration of 2 µM. The assay was carried out under saturating concentrations of ATP (2 mM) using ctXPD wild type and variants at a concentration of 500 nM with ctp44 in a 1∶2 stoichiometric ratio as indicated. Prior to the analysis, ctXPD was heated to 50°C. For catalytic measurements, the mix of all reagents, with the exception of ATP, was preincubated at 37°C until a stable base line was achieved. Enzyme catalysis was initiated by the addition of ATP. The activity profiles were measured at 340 nm using a Floustar Optima plate reader. Initial velocities were recorded and ATP consumption was determined using the molar extinction coefficient of NADH. The measurements were carried out in triplicates and with at least two different protein batches.

### *In Vitro* Helicase Assay

Helicase activity was analyzed utilizing a fluorescence-based helicase assay [8],[37]. We used a 5′ overhang substrate with a cy3 label at the 3′ end of the translocated strand (5′-agctaccatgcctgcacgaattaagcaattcgtaatcatggtcatagct-3′-cy3) and a dabcyl modification on the 5′ end of the opposite strand (Dabcyl-5′-agctatgaccatgattacgaatt-3′). This results in a quenching of the cy3 fluorescence that is removed upon unwinding of the substrate. Assays were carried out in 20 mM Tris-HCl pH 8.0, 10 mM KCl, 1 mM MgCl_2_, and 1 mM TCEP. ctXPD wild type and variants were used at a concentration of 500 nM, with concentrations of ctp44 in a 1∶2 molar ratio where indicated. Prior to the analysis, ctXPD was heated to 50°C. The proteins were mixed with 40 nM open fork substrate and 250 nM capture oligonucleotide (5′-caattcgtaatcatggtc-3′). The reaction was subsequently started with the addition of 2 mM ATP. Kinetics were recorded with a Flouromax4 fluorescence spectrometer (Horiba Jobin Yvon) and monitored until the reaction was completed, where possible. Fluorescence was detected at an excitation wavelength of 550 nm (slid width, 2 nm) and an emission wavelength of 570 nm (slid width, 2 nm). Initial velocities were fitted with Origin8 and represent the averages of at least two different reactions and two independent protein batches.

### Construction of Baculoviruses, Protein Production, and Purification

The cDNAs encoding full-length hsXPD wild type and variants (C134S, C155S, Y158A, F161A, F193A, R196A, R196E, L372A, and R722) were cloned into pAC8F [11],[12],[49]. Resulting transfer vectors were recombined with baculovirus DNA (BaculoGold DNA, Pharmingen) in *Sf*9 cells to generate viruses for production of hsXPD in fusion with the Flag peptide (DYKDDDDK).

For production of proteins and complexes, *Sf*21 cells were infected/coinfected with the appropriate viruses/combinations of viruses, collected 48 h postinfection, and purified as described [32], Briefly, cells resuspended in buffer A (20 mM Tris–HCl, pH 8.0, 250 mM KCl, and 1 mM DTT) containing complete protease inhibitor cocktail (Roche) were disrupted by sonication, and after clarification, the lysate was incubated with protein A sepharose beads cross-linked to the M2 anti-Flag antibody (SIGMA™) for purification of XPD or CAK (both harbor an epitope FLAG) and to the 1H5 anti-p44 antibody for purification of core-IIH. After extensive washing in buffer A and equilibration in buffer B (50 mM Tris-HCl pH 8.0, 75 mM KCl, 20% glycerol, 0.1% NP-40, and 1 mM DTT), proteins were eluted by competition with 2 CV of buffer B containing the appropriate synthetic peptide at 0.5 mg/ml. Protein concentrations were estimated by quantitative WB analysis and adjusted to ∼100 ng/µl. Monoclonal antibodies against the human TFIIH subunits XPB (1B3), XPD (2F6), p52 (1D11), p44 (1H5), CDK7 (2F8), MAT1 (2D3), and p8 (1D1) were obtained from IGBMC's facilities.

### Helicase and Dual Incision Assays

The 5′-3′ hsXPD helicase activity was analyzed using a mono-directional strand displacement assay (for review, see [8],[23]). The probe was prepared by mixing an oligonucleotide (5 ng) corresponding to nucleotides 6228–6251 of the single-stranded M13mp18(+) DNA with single-stranded M13mp18(−) phage (1 mg) in the presence of NaCl (25 mM) and MgCl2 (2.5 mM). The mixture was heated for 2 min at 100°C and cooled slowly to RT to allow annealing of the DNA heteroduplex. Probe labeling was performed using the Klenow fragment in the presence of dTTP (50 mM) and [α^32^P]dATP (70 µCi; 3,000 Ci/mmol; Amersham). After phenol/chloroform extraction, the labeled probe was purified using Micro Bio-Spin clean-up columns (Biorad™).

Equivalent amounts of purified hsXPD wild type and variants (∼200 ng) were added to a 5′-strand extension probe in the presence of a 1.5 molar excess of p44 (∼150 ng) to evaluate their 5′–3′ helicase activity. The reaction was performed for 45 min at 37°C by adding the immunopurified helicase to the DNA probe at 10 nM in 20 mM Tris-HCl (pH 8.0), 75 mM KCl, 4 mM MgCl_2_, 1 mM DTT, 4 mM ATP, and 0.1 mg/ml BSA with a total reaction volume of 25 µl. The reaction was stopped by adding 20 mM EDTA, 14% glycerol, 0.2% SDS, and 0.028% bromophenol to the reaction mixture. Analyses were performed by migration in a 14% polyacrylamide gel (acrylamide/bis-acrylamide ratio, 33/1) and autoradiography.

NER dual incision assays were performed as described [20]–[22],[26] using a plasmid with a single 1,3-intrastrand d(GpTpG) (30 ng) in a buffer containing 50 mM Hepes-KOH (pH 7.8), 5 mM MgCl_2_, 1 mM DTT, 0.3 mM EDTA, 10% glycerol, 2.5 µg BSA, 50 mM KCl, and 2 mM ATP. Reaction mixes (25 µl) containing human XPG (5 ng), XPF/ERCC1 (15 ng), XPC/hHR23B (10 ng), RPA (50 ng), XPA (25 ng), as well as a mixture of purified core-IIH (250 ng) and XPD (100 and 200 ng) were assembled to reconstitute TFIIH activity. Mixes were incubated at 30°C for 90 min and analyzed as previously described.

### Dual-Luciferase Reporter Assay

The pGL3-SV40 vector expressing the FireFly luciferase under the control of the SV40 promoter was UV irradiated (254 nm, 1,000 J/m^2^) at a concentration of 100 ng/µl in 10 mM Tris-HCl (pH 8.0) and 1 mM EDTA. HD2 fibroblasts were seeded in DMEM/HAM-F10 containing 10% FCS and 10 µg/ml gentamcin in a 24-well plate (50,000 cells/well). After 20 h, each well was co-transfected using JetPEI (Polyplus) with 450 ng pGL3-SV40 (UV+/), 100 ng of pRL-TK expressing Renilla luciferase under the control of the thymidine kinase promoter to normalize transfection efficiencies, and 25 ng of pIRES2-EGFP derivatives for expression of hsXPD wild type or variants under the control of the CMV promoter and 450 ng of pBS. The medium was exchanged after 3 h and further incubated for 36 h. Cell lysates were prepared and activities were assayed using the Dual-Glo Luciferase assay system (Promega).

### *In Vitro* Transcription and Phosphorylation Assays

Run-off transcription assays were performed as described [12],[24],[25] except that TFIIH was substituted by a mixture of purified core-IIH, CAK, and XPD, which allowed us to prepare a pre-mix containing all components with the exception of the XPD variants. Reaction mixes contained the adenovirus major late promoter sequence (AdMLP) EcoRI–SalI DNA template (75 ng), TFIIB (15 ng), TFIIE (160 ng), TFIIF (500 ng of the phenyl fraction from Pol II and the GTF purification scheme), TBP (30 ng), endogenous RNAP II (10 µg of the 1 M DEAE fraction), and a mixture of the purified core-IIH, CAK, and XPD. RNAP II phosphorylation was carried out as a classical runoff transcription except that ATP was added to a final concentration of 5 mM, and the amount of purified RNAP II polymerase was adjusted (typically reduced by a factor of 3). Hypo (IIA) and hyper (IIO) phosphorylated forms of RNAP II were resolved on a 6% SDS-PAGE and detected by Western blot using the monoclonal antibody (7C2) directed against the CTD.

## Supporting Information

Figure S1 **Human and ctXPD.** (a) Sequence alignment of human and ctXPD. Identical residues are shown in white with a red background. The different variants are indicated with a blue dot and labeled; every 10^th^ residue is marked by a small black dot. Residues marked in red surrounded by a blue box are not strictly conserved but indicate similar residues in close proximity that were not captured by the sequence alignment. (b) Structure of taXPD and the location of the different variants depicted as cpk models. The schematic representation of the C terminus of eukaryotic XPD that interacts with p44 is shown as dark red oval.
(TIF)

Figure S2 **Stability analysis of ctXPD and its variants.** (a) Coomassie stained gel of ctXPD wild type and its variants. Only the C133S variant proved to be less stable than the wild-type protein during purification. (b) CD spectroscopy of ctXDP and its variants. The analysis by CD spectroscopy clearly shows that all the XPD variants assume the same fold as the wild-type protein. (c) SEC of different ctXPD variants in the presence of p44. Each variant is indicated in the chromatograms. XPD and p44 were individually analyzed and are shown in blue and pink, respectively, whereas the complex analysis is shown in red.
(TIF)

Figure S3 **Interaction of XPD, p44, and MAT1.** (a) Native PAGE analysis of ctXPD and its variants showing the interaction between ctXPD and ctMAT1. The different protein samples are indicated above the respective lanes. Native PAGE analysis was carried out using 6% Tris-glycine gels pH 8.1, and 500 nM wild-type ctXPD (or the indicated variant) were incubated with equimolar amounts of ctMAT1. The second band present in the ctMAT1 sample has been identified by mass spectroscopy as a degradation product of ctMAT1 lacking the N-terminal RING domain of MAT1(1–83). (b and c) Pairwise interactions between hsXPD variants and hsCAK or hsp44. Purified wild-type or mutated human XPD variants were mixed with CAK or p44 in a buffer containing 150 mM NaCl, 0.1% Nonidet P-40, 1 mM DTT, 50 mM Tris/HCl at pH 8, and incubated for 4 h at 4°C in the presence of anti-XPD antibody cross-linked to Protein A agarose beads (5 µl per experiment). After extensive washing, immunoprecipitated complexes were resolved by SDS/PAGE with 12% (wt/vol) polyacrylamide and detected by Western blot using anti-Cdk7, anti-cyclin H, or anti-p44 monoclonal antibodies. The asterisks indicate the antibody light (LH) and heavy chains (HC).
(TIF)

Figure S4 **Time-dependent transcriptional activity of hsXPD and selected variants.** Recombinant wild-type and mutant C134S, R722W, and K48R XPD variants (∼100 ng) were mixed with purified core-TFIIH (rIIH6) (250 ng) and CAK (300 ng) and added to an *in vitro* reconstituted transcription system containing all the basal transcription factors and the adenovirus major late promoter sequence (AdMLP) EcoRI–SalI DNA template (lanes 1–15). Following 10, 20, and 45 min incubation at 30°C (lanes 2–14), transcripts were analyzed by electrophoresis followed by autoradiography. A transcription experiment performed with endogenous TFIIH was included as a control (lane 15). ^32^P-labelled pBR322/Msp1 digests were used as size markers (lane 16). The data used to generate the lower panel have been deposited as supplementary information in xls format (Table S4).
(TIF)

Table S1 **Contains data for** **Figure 2** **.**
(XLSX)

Table S2 **Contains data for** **Figure 3** **.**
(XLSX)

Table S3 **Contains data for** **Figure 4** **.**
(XLSX)

Table S4 **Contains data for Figure S4.**
(XLSX)

## Abbreviations

NER: nucleotide excision repair
SF: superfamily
TFIIH: transcription factor II H
XPB: xeroderma pigmentosum variant B
XPD: xeroderma pigmentosum variant D
