# Supplementary figures and images for: In TFIIH, XPD Helicase Is Exclusively Devoted to DNA Repair
Source: PLoS Biol. 2014 Sep 30;12(9):e1001954. doi: 10.1371/journal.pbio.1001954 (PMC4182028; doi:10.1371/journal.pbio.1001954)

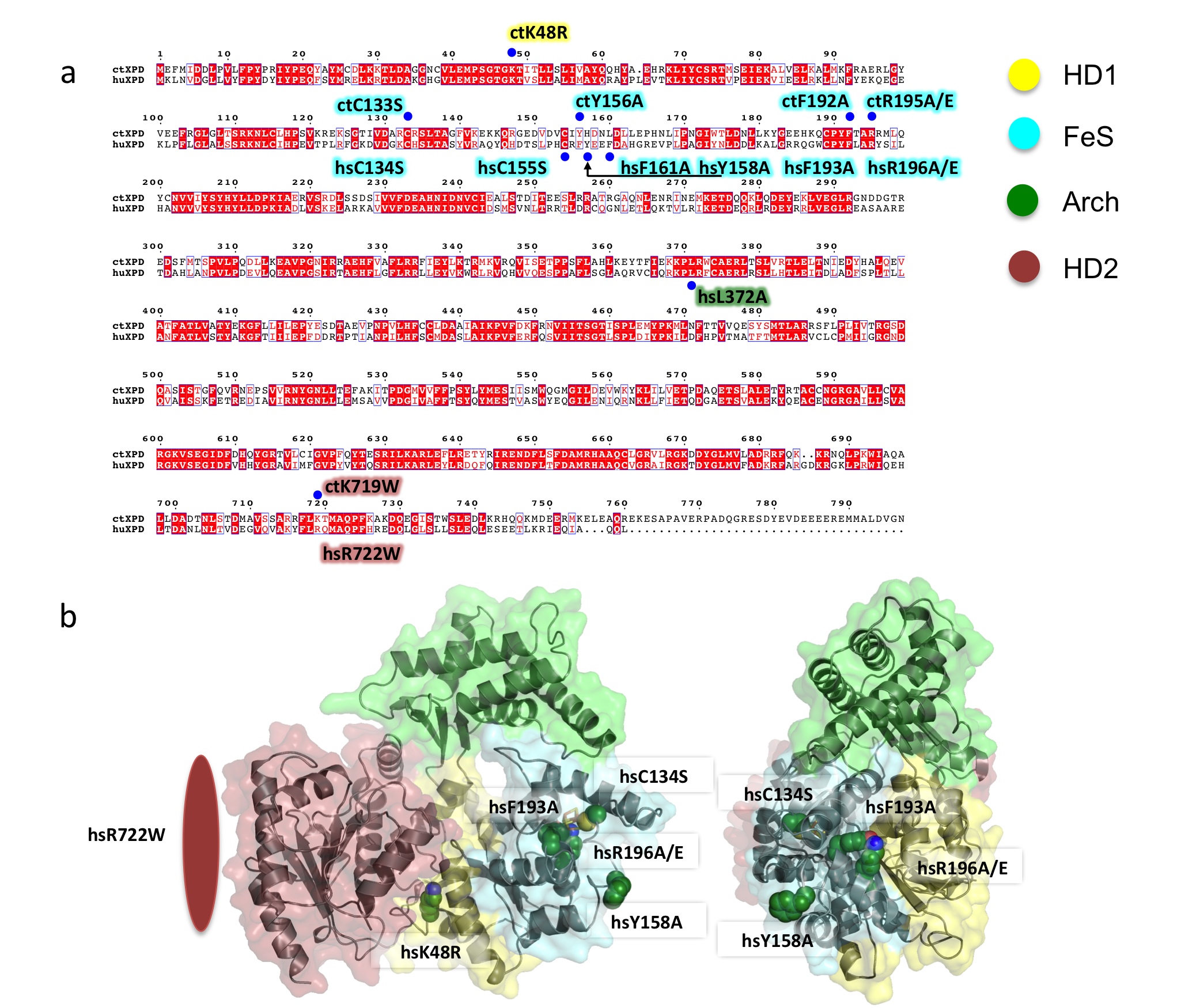

Supplement: Figure S1 — Human and ctXPD. (a) Sequence alignment of human and ctXPD. Identical residues are shown in white with a red background. The different variants are indicated with a blue dot and labeled; every 10th residue is marked by a small black dot. Residues marked in red surrounded by a blue box are not strictly conserved but indicate similar residues in close proximity that were not captured by the sequence alignment. (b) Structure of taXPD and the location of the different variants depicted as cpk models. The schematic representation of the C terminus of eukaryotic XPD that interacts with p44 is shown as dark red oval. (TIF) [file pbio.1001954.s001.tif]

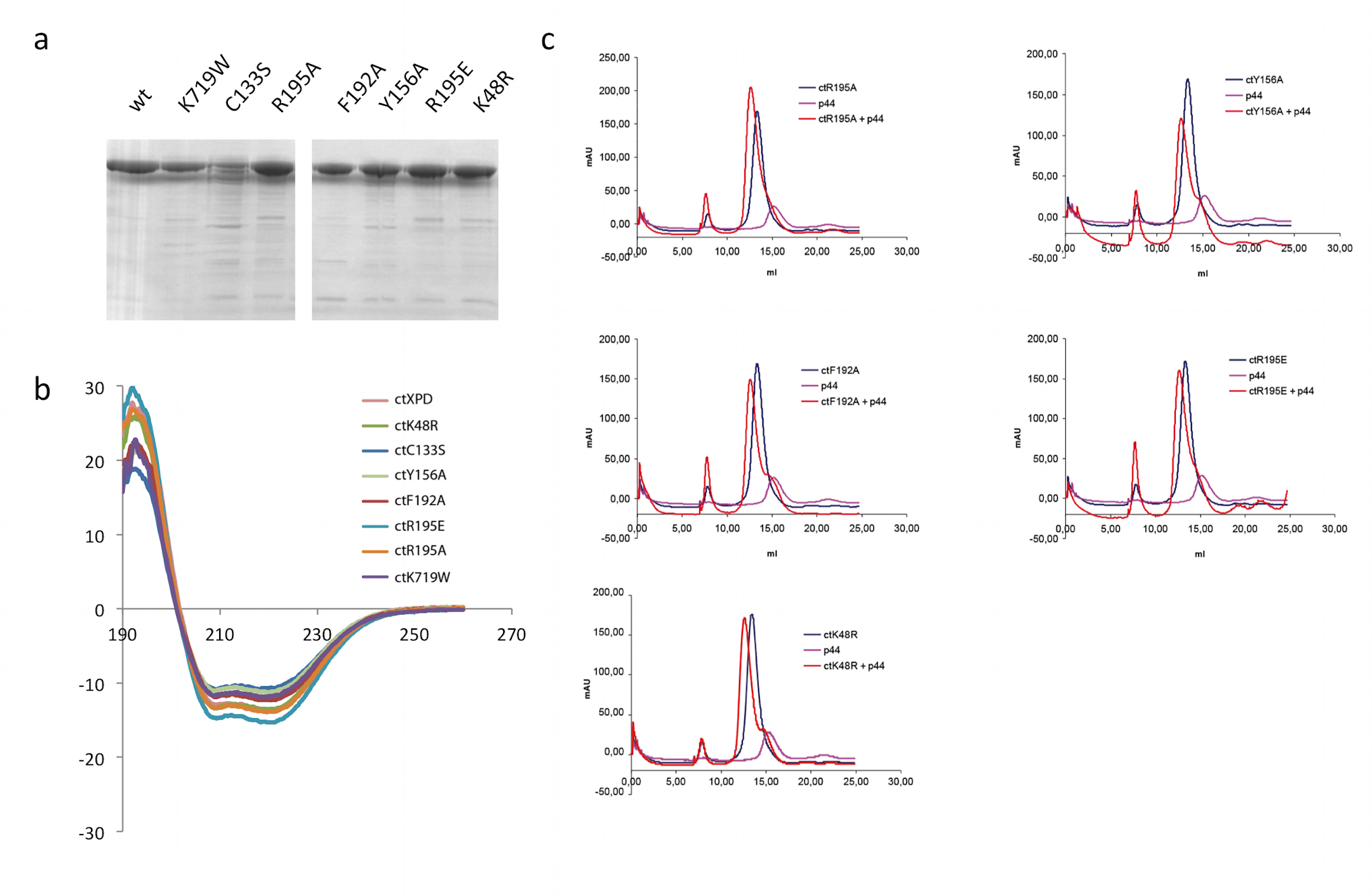

Supplement: Figure S2 — Stability analysis of ctXPD and its variants. (a) Coomassie stained gel of ctXPD wild type and its variants. Only the C133S variant proved to be less stable than the wild-type protein during purification. (b) CD spectroscopy of ctXDP and its variants. The analysis by CD spectroscopy clearly shows that all the XPD variants assume the same fold as the wild-type protein. (c) SEC of different ctXPD variants in the presence of p44. Each variant is indicated in the chromatograms. XPD and p44 were individually analyzed and are shown in blue and pink, respectively, whereas the complex analysis is shown in red. (TIF) [file pbio.1001954.s002.tif]

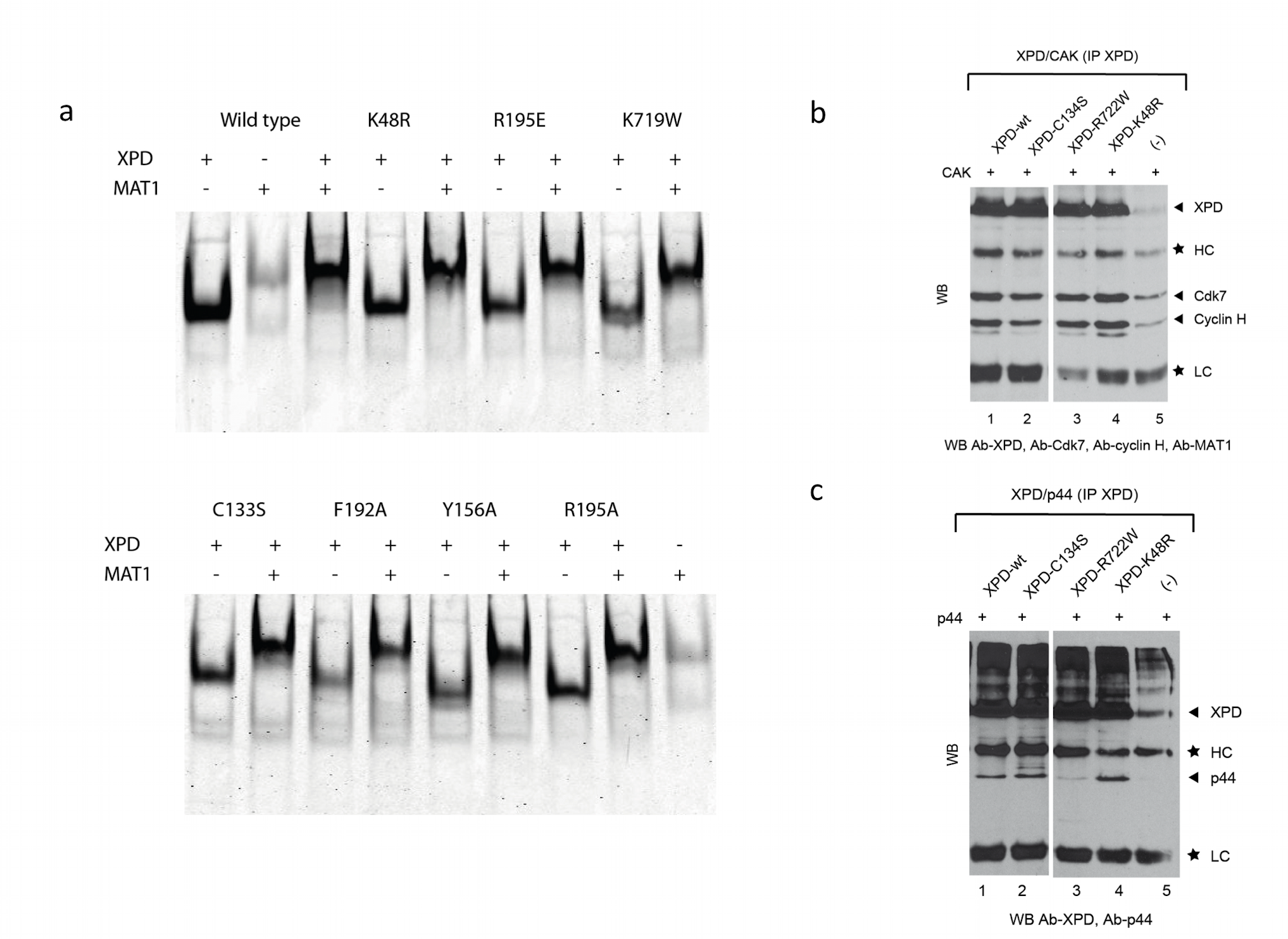

Supplement: Figure S3 — Interaction of XPD, p44, and MAT1. (a) Native PAGE analysis of ctXPD and its variants showing the interaction between ctXPD and ctMAT1. The different protein samples are indicated above the respective lanes. Native PAGE analysis was carried out using 6% Tris-glycine gels pH 8.1, and 500 nM wild-type ctXPD (or the indicated variant) were incubated with equimolar amounts of ctMAT1. The second band present in the ctMAT1 sample has been identified by mass spectroscopy as a degradation product of ctMAT1 lacking the N-terminal RING domain of MAT1(1–83). (b and c) Pairwise interactions between hsXPD variants and hsCAK or hsp44. Purified wild-type or mutated human XPD variants were mixed with CAK or p44 in a buffer containing 150 mM NaCl, 0.1% Nonidet P-40, 1 mM DTT, 50 mM Tris/HCl at pH 8, and incubated for 4 h at 4°C in the presence of anti-XPD antibody cross-linked to Protein A agarose beads (5 µl per experiment). After extensive washing, immunoprecipitated complexes were resolved by SDS/PAGE with 12% (wt/vol) polyacrylamide and detected by Western blot using anti-Cdk7, anti-cyclin H, or anti-p44 monoclonal antibodies. The asterisks indicate the antibody light (LH) and heavy chains (HC). (TIF) [file pbio.1001954.s003.tif]

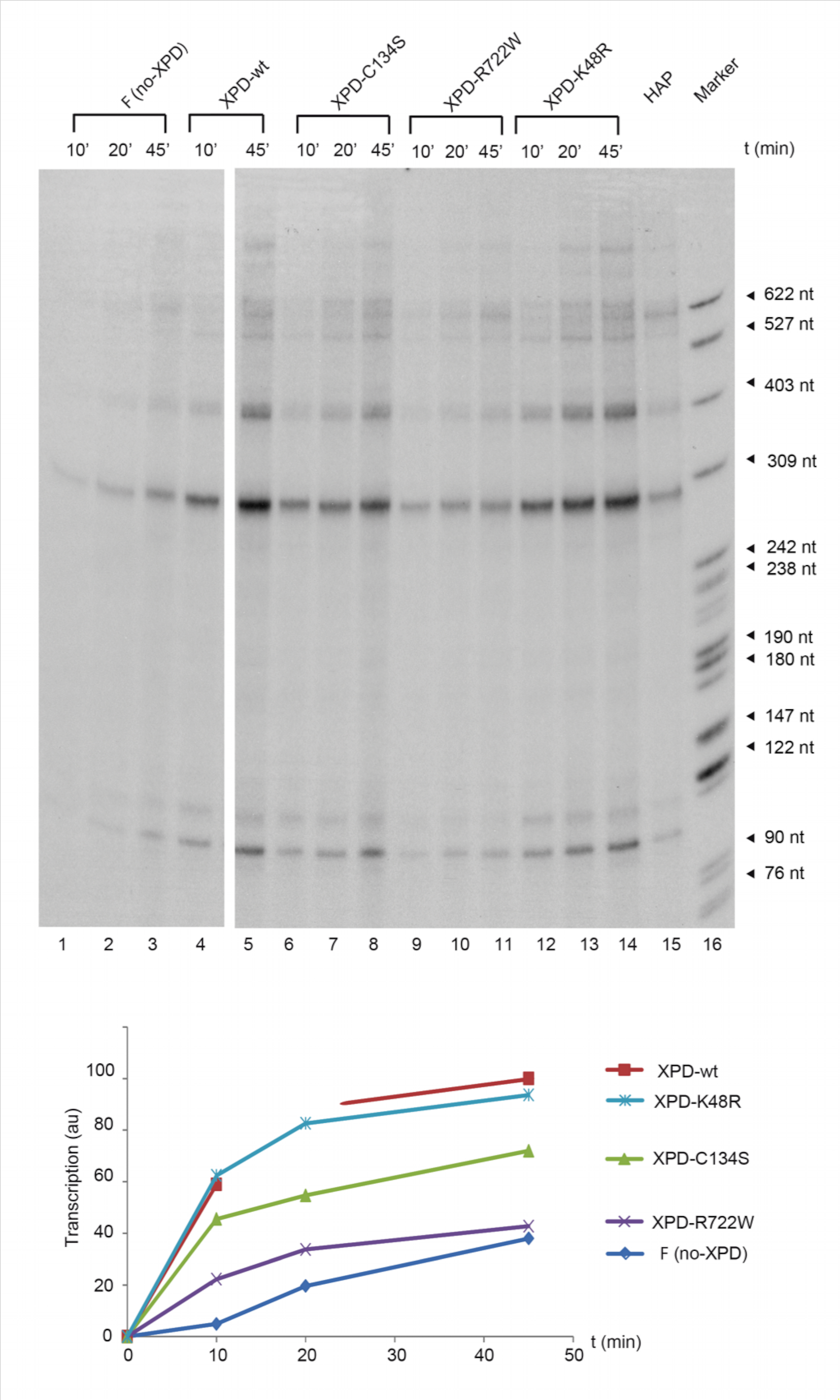

Supplement: Figure S4 — Time-dependent transcriptional activity of hsXPD and selected variants. Recombinant wild-type and mutant C134S, R722W, and K48R XPD variants (∼100 ng) were mixed with purified core-TFIIH (rIIH6) (250 ng) and CAK (300 ng) and added to an in vitro reconstituted transcription system containing all the basal transcription factors and the adenovirus major late promoter sequence (AdMLP) EcoRI–SalI DNA template (lanes 1–15). Following 10, 20, and 45 min incubation at 30°C (lanes 2–14), transcripts were analyzed by electrophoresis followed by autoradiography. A transcription experiment performed with endogenous TFIIH was included as a control (lane 15). 32P-labelled pBR322/Msp1 digests were used as size markers (lane 16). The data used to generate the lower panel have been deposited as supplementary information in xls format (Table S4). (TIF) [file pbio.1001954.s004.tif]
